# Redefining Hematology Referral Thresholds for Eosinophilia

**DOI:** 10.1111/ejh.70152

**Published:** 2026-03-15

**Authors:** Payal Bharatkumar Patel, Corinne LaVasseur, Parmis Sahrapima, Riley Meister, Thalia Padilla Kelley, Lukas Seifer, Thomas Deloughery, Joseph J. Shatzel

**Affiliations:** 1 Department of Medicine, Legacy Medical Center, Portland, Oregon, USA; 2 Division of Hematology and Medical Oncology, Oregon Health & Science University, Portland, Oregon, USA; 3 Division of Medicine, Oregon Health & Science University, Portland, Oregon, USA; 4 Department of Biomedical Engineering, Oregon Health & Science University, Portland, Oregon, USA

**Keywords:** absolute eosinophilia count, eosinophilia, hypereosinophilic syndrome

## Abstract

**Objectives:**

Eosinophilia is a common laboratory finding that frequently prompts referral for hematologic evaluation, yet the clinical utility of these consultations remains poorly characterized. This study aims to refine stratification for eosinophilia and characterize the spectrum of associated diagnoses.

**Methods:**

We conducted a retrospective study of patients referred to hematology at the Oregon Health & Science University between 2015 and 2025 with confirmed eosinophilia based on an absolute eosinophil count (AEC) ≥ 0.5 × 10^9^/L.

**Results:**

A total of 142 patients were evaluated for eosinophilia during the study period. The etiology of eosinophilia was deemed idiopathic in 44.8% (*n* = 64) of patients. Hypereosinophilic syndrome was diagnosed in 20.3% of patients (*n* = 29) and analyzed as a clinical syndrome, with most cases classified as HES not otherwise specified. The identifiable secondary causes of eosinophilia included asthma (9.8%), parasitic infection (7.7%), autoimmune disease (4.9%), and drug reactions (4.9%). Eosinophilia-specific therapies were administered in 22.5% (*n* = 32) of patients. Receiver operating characteristic (ROC) analysis demonstrated that peak AEC was a strong predictor of clinically meaningful eosinophilia, defined as a diagnosis of HES or need for eosinophilia-specific treatment, with an area under the curve of 0.83. An AEC threshold of 1.5 × 10^9^/L yielded a high sensitivity of 97% for clinically meaningful eosinophilia.

**Conclusions:**

These findings support considering a threshold of 1.5 × 10^9^/L or higher for hematology referral, especially in low-risk and asymptomatic patients, though external validation is needed.

## 1 | Introduction

Eosinophilia is a frequent referral in classical hematology; however, the broad differential diagnosis, diverse clinical implications, and paucity of evidence-based guidance make evaluation challenging. One of the hematologist’s main concerns is distinguishing clinically significant, potentially harmful eosinophilia from secondary or benign reactive causes. Eosinophilia may result from clonal expansion of eosinophil precursors due to somatic hematopoietic mutations, whereas secondary eosinophilia is reactive, arising in response to cytokine-driven proliferation triggered by nonneoplastic and paraneoplastic processes [1, 2]. The latter may occur in association with a wide range of underlying conditions, including infections, allergic or autoimmune diseases, asthma, pulmonary disorders, and malignancies, resulting in a broad differential diagnosis [3, 4]. Additionally, a large subset of patients present with idiopathic eosinophilia, occurring when neither a clonal nor a reactive cause can be identified [5, 6]. Lastly, hypereosinophilic syndrome (HES) involves eosinophilic tissue infiltration with end-organ damage and, like eosinophilia, may be primary, secondary, or idiopathic [7–9]. Despite advances in diagnostic testing, the evaluation and management of eosinophilia remain challenging, underscoring the need for an evidence-based diagnostic approach.

Eosinophilia frequently prompts referral to hematology, even though eosinophilia is more often caused by nonhematologic conditions, such as allergic, infectious, or inflammatory diseases [10, 11]. In many cases, the eosinophilia itself is asymptomatic and discovered incidentally on routine laboratory testing, leading to uncertainty regarding the appropriate extent of diagnostic evaluation [12, 13]. Current guidelines offer limited direction on how to stratify patients by eosinophil count, duration, or accompanying clinical features to determine when subspecialty referral or advanced testing is warranted [14]. As a result, clinical practice varies widely, ranging from serial observation to extensive and sometimes unnecessary hematologic investigations [15].

This study aims to address these gaps by characterizing the relationship between eosinophil count parameters and clinically relevant findings, including the presence of organ involvement and need for subspecialist-directed therapy. Defining such correlations may help refine diagnostic algorithms, optimize referral patterns, and reduce the burden of unnecessary testing while ensuring timely identification of patients requiring targeted evaluation and treatment.

## 2 | Methods

### 2.1 | Study Design

We conducted a retrospective study of all patients (ages 18 and older) referred to the Oregon Health and Science University (OHSU) outpatient hematology department for initial evaluation of eosinophilia from 2015 to 2025. This study was approved by the OHSU institutional review board before initiation (STUDY00027938).

### 2.2 | Participants

Eligibility criteria required: (1) diagnosis of eosinophilia by ICD code (D72.10, D72.19, D72.1) and (2) referral to the OHSU hematology clinic for initial evaluation of eosinophilia. Exclusion criteria included individuals who were not evaluated by OHSU hematology after referral, had a normal eosinophil count at the time the referral was placed (< 0.5 × 10^9^), and/or had a prior evaluation and/or treatment for eosinophilia by an outside hematologist at the time of OHSU referral. Data collection included age, race, ethnicity, medical history, laboratory studies, eosinophil-related diagnosis, and treatment.

### 2.3 | Outcomes

The primary outcome of the study was clinically meaningful eosinophilia, defined as a diagnosis of HES or the need for eosinophilia-specific treatment, characterized as treatment with prednisone, mepolizumab, hydroxyurea, imatinib, pegylated interferon, and/or mycophenolate for an indication of or symptoms due to eosinophilia. HES was defined as peripheral blood eosinophilia of at least 1.5 × 10^9^/L or confirmed tissue infiltration of eosinophils on biopsy and evidence of end-organ damage attributable to eosinophilia.

Secondary outcomes of interest included systemic treatment for secondary causes of eosinophilia, including asthma, parasitic infections, and autoimmune disease, as well as time to normalization of eosinophil count.

### 2.4 | Data Analysis

Descriptive statistics were performed to summarize baseline characteristics and outcome data, with categorical variables presented as a number and percentage, and numerical variables presented as means and standard deviation. AEC was used as the primary analytic variable due to variability in total leukocyte counts across patients. Eosinophil percentage was collected and included as a descriptive variable. Receiver operating characteristic (ROC) analysis was performed and Youden’s index was generated to measure the diagnostic effectiveness of eosinophil count and identify the optimal threshold for combined sensitivity and specificity. Additionally, positive and negative predictive values (PPV, NPV) were measured at this optimal threshold. Kaplan–Meier analysis was used to evaluate the time to normalization of eosinophil count. Analyses were performed using SPSS 30.0.0.0, and figures were generated with GraphPad Prism 10.6.0.

## 3 | Results

In total, 215 patients were identified. Seventy-three patients were excluded from the study (Figure 1). The included cohort of 142 patients was predominantly female (65%, *n* = 92) (Table 1). At the time of referral, 45 patients (31.7%) had mild eosinophilia (0.5–1.5 × 10^9^/L), 71 patients (50%) had moderate eosinophilia (1.5–5.0 × 10^9^/L), and 26 patients (18.3%) had severe eosinophilia (> 5.0 × 10^9^/L). The eosinophil count at the time of referral ranged from 0.5 to 45.66 × 10^9^/L. The mean time from hematology referral to clinic evaluation was 124 days (approximately 4.1 months). At the time of hematology appointment, 18.3% (*n* = 26) had spontaneous resolution of eosinophilia (AEC ≤ 0.5 × 10^9^/L). These patients generally had lower peak AECs and predominantly reactive or idiopathic etiologies. Detailed characteristics of this subgroup, including peak AEC, eosinophil percentage, and final diagnosis, are provided in Table S1. Across the entire cohort, the etiology of eosinophilia was deemed idiopathic in 44.8% of patients (*n* = 64). Identifiable secondary etiologies included asthma (9.8%, *n* = 14), parasitic infection (7.7%, *n* = 11), autoimmune disease (4.9%, *n* = 7), allergic (4.9%, *n* = 7), drug-related reactions (4.9%, *n* = 7), and gastrointestinal disease (2.1%, *n* = 3). Allergic etiologies included allergic rhinitis and eczema, while gastrointestinal causes comprised eosinophilic esophagitis and eosinophilic gastroenteritis confirmed by endoscopic and histopathologic evaluation. Infectious etiologies were limited to parasitic infections, including *Strongyloides* and *Dientamoeba fragilis*. Drug-related eosinophilia was most frequently associated with antibiotics, nonsteroidal anti-inflammatory drugs, and anticonvulsants. 20.3% of patients (*n* = 29) met criteria for HES. HES was considered a clinical syndrome rather than a distinct etiologic diagnosis; therefore, these cases were further subclassified according to underlying cause when identifiable (Tables 1 and 2A,B).

### 3.1 | Primary Outcomes

Clinically meaningful eosinophilia, defined as diagnosis of HES or utilization of eosinophilia-specific treatment, occurred in 35 patients (25.4%; Table 2B). The peak AEC in patients with clinically meaningful eosinophilia ranged from 1.36–45.66 × 10^9^/L with a mean AEC of 11.33 × 10^9^/L.

Twenty-nine patients met criteria for HES, defined as peripheral blood eosinophilia of at least 1.5 × 10^9^/L or tissue infiltration of eosinophils along with evidence of end-organ damage attributable to the eosinophilia. Peak AEC ranged from 1.51 to 45.66 × 10^9^/L with an mean of 34.5 × 10^9^/L. HES was considered a clinical syndrome rather than an etiologic diagnosis, and cases were subclassified according to underlying cause when identifiable. The majority of HES cases were classified as HES not otherwise specialized (NOS) in 65.5% of cases (*n* = 19). The other etiologies of HES were autoimmune disease (13.85%, *n* = 4), hematologic malignancy/myeloproliferative neoplasm (6.9%, *n* = 2), overlap syndromes (6.9%, *n* = 2), allergy (3.45%, *n* = 1), and eosinophilic pneumonia (3.45%, *n* = 1) (Table 2A).

Eosinophilia-specific treatment with prednisone, hydroxyurea, mepolizumab, imatinib, pegylated interferon, and mycophenolate was administered to 34 patients (24%, Table 3). The indication for treatment in all cases included eosinophilia, with a peak AEC range of 1.6–45.66 × 10^9^/L (mean 10.9 × 10^9^/L). Five patients received treatment for eosinophilia without a diagnosis of HES (Table 2B). The peak AEC of this group ranged from 1.7 to 18.08 × 10^9^/L.

### 3.2 | Secondary Outcomes

An additional 26 patients received systemic treatment for secondary causes of eosinophilia, including asthma, parasitic infections, and autoimmune disease. The most common treatment was ivermectin for documented parasitic infection (*n* = 9). The range for AEC in this patient population was 0.8–21.7 × 10^9^ (mean: 5.18 × 10^9^/L) (Table S2).

The mean time to normalization, defined as an AEC < 0.5 × 10^9^/L, among patients with available follow-up data was 426 days (approximately 14 months) through 2025. Of the 142 patients, normalization occurred in 62.7% (*n* = 89) patients, while 37.3% (*n* = 53) did not achieve normalization. Among the 60 patients who required treatment, 63.3% (*n* = 38) achieved normalization. Of the 82 patients who were not treated, eosinophil counts normalized in 62.2% (*n* = 51) and remained > 0.5 × 10^9^/L in 37.8% (*n* = 31). Patients who received systemic or eosinophilia-specific therapy achieved normalization more rapidly, with a median time of 8.5 months, compared with 12.6 months among those who did not receive treatment. There was no statistically significant difference between the median time to normalization in these groups (log-rank *x*^2^ = 0.38, *p* = 0.54, Figure S1).

### 3.3 | Sensitivity and Specificity Analysis

ROC analysis demonstrated that peak absolute eosinophil count (AEC) for clinically meaningful eosinophilia had an area under the curve of 0.83 (Figure 2). An AEC threshold of 1.5 × 10^9^/L yielded a 97% sensitivity for clinically meaningful eosinophilia and a specificity of 41%. This threshold also resulted in a 97% sensitivity for eosinophilia-specific treatment and a 92% sensitivity for any systemic treatment.

The Youden’s index (0.493) for clinically meaningful eosinophilia occurred at an AEC of 2.11 × 10^9^/L (sensitivity of 89%, specificity of 61%). The PPV and NPV for clinically meaningful eosinophilia at an AEC of 2.11 × 10^9^/L were 42.7% and 94.4%, respectively (Figure 3). The optimal thresholds were identified for eosinophilia-specific treatment (2.11 × 10^9^/L, sensitivity 82%, specificity 63%) and any systemic treatment (2.40 × 10^9^/L, sensitivity 78%, specificity 67%).

## 4 | Discussion

This study evaluates the diagnostic performance of AEC thresholds within a real-world hematology referral population. Our objective was to refine a threshold for clinically meaningful eosinophilia and delineate associated diagnoses with a focus on primary hematologic conditions. This approach was designed to identify patients most likely to benefit from hematologic evaluation and treatment, while minimizing unnecessary investigations in those with transient eosinophilia.

In our cohort, a peak AEC of 1.5 × 10^9^/L demonstrated a high sensitivity (97%) for detecting clinically meaningful eosinophilia, defined as diagnosis of HES or need for eosinophilia-specific treatment. The optimal threshold determined by Youden’s index was 2.11 × 10^9^/L, though this level resulted in a modest reduction in sensitivity (89%), increasing the likelihood of missed cases. Only two of the 142 patients were diagnosed with a primary hematologic disorder (T-cell lymphoma and essential thrombocythemia), both of which had HES with eosinophil counts > 1.5 × 10^9^/L. Collectively, our results suggest that adopting a higher eosinophil threshold for hematology referral, such as 1.5 × 10^9^/L, may improve diagnostic efficiency and limit unnecessary consultation without compromising sensitivity for detecting clinically significant disease. However, this potential benefit should be interpreted cautiously given the modest specificity observed at this threshold.

The severity of eosinophilia is regularly categorized based on the AEC: mild (0.5–1.5 × 10^9^/L), moderate (1.5–5.0 × 10^9^/L), and severe (> 5.0 × 10^9^/L) [16]. Notably, these thresholds do not reliably correlate with clinical severity or underlying etiology [10]. Nearly one-third of patients (31%) in our study were referred to and evaluated by hematology for mild eosinophilia (peak AEC 0.5–1.5 × 10^9^/L), which is below our threshold we identified for clinically meaningful eosinophilia (1.5 × 10^9^). Eosinophilia also resolved in 18% of patients by the time of their hematology appointment. We observed similar time-to-normalization patterns regardless of treatment in our study, with resolution of eosinophilia in 62.2% (*n* = 51) of patients without intervention compared to 63% (*n* = 38) of those who received treatment for primary or secondary causes of eosinophilia. Overall, these findings indicate that a substantial proportion of eosinophilia cases are mild and self-limited. Importantly, the median interval of approximately 4 months between referral and hematology evaluation likely contributed to the observed rate of spontaneous normalization of eosinophilia. This delay reflects real-world access to subspecialty care at a tertiary academic center and may allow transient or reactive eosinophilia to resolve without intervention.

Our results reinforce the current clinical guidelines, which suggest treatment should be reserved for patients who are symptomatic, exhibit evidence of organ involvement, or have persistently elevated eosinophil counts [17, 18]. They also support a surveillance-based approach for asymptomatic patients with mild–moderate eosinophilia, as additional testing in this group is unlikely to change management. Broad application of these criteria may minimize unnecessary testing and treatment exposure while ensuring appropriate care for patients at the highest risk of eosinophil-mediated complications.

Our study has several strengths, including individual patient chart review, detailed stratification by peak AECs, and long-term follow-up extending up to a decade, allowing for a robust assessment of outcomes. Limitations include the retrospective design, which limited our ability to standardize assessment of symptom burden, duration of eosinophilia, and organ involvement, reducing our ability to fully distinguish between asymptomatic and symptomatic eosinophilia with full accuracy. The study was conducted at a single academic center and included a predominantly Caucasian population, which may limit generalizability to more racially, ethnically, and practice-diverse settings. Our study population could also under-represent certain hematologic diagnoses, as we excluded patients previously evaluated by a hematologist or referred for a second opinion. In community practice, patients with clinically mild or transient eosinophilia may be managed without referral, while others may receive treatment prior to hematology evaluation, potentially introducing referral and selection bias. Treatment decisions were partially influenced by AEC, which also served as the primary predictor in diagnostic performance analyses, potentially inflating the apparent discriminative ability of AEC thresholds. The low prevalence of myeloid neoplasms in this cohort, including PDGFRA/PDGFRB-rearranged disease, limits conclusions regarding referral thresholds in disorders where eosinophilia may be mild yet clinically significant. Although clinical significance is influenced by factors such as symptoms, disease duration, organ involvement, and comorbidities, we did not perform multivariable modeling incorporating these variables. Given the limited number of outcome events and the high correlation between these features and AEC, inclusion of multiple predictors risked overfitting and misinterpretation. Diagnostic testing was comprehensive in many cases; however, not all patients underwent bone marrow biopsy, which is one of the most sensitive evaluations for clonal hematopoietic disorders [19]. Additionally, treatment approaches were not standardized, limiting conclusions about the comparative efficacy of individual regimens. Finally, while our diagnostic accuracy analyses provide practical thresholds for referral, these cutoffs should be externally validated in larger, more diverse cohorts before being universally adopted.

## 5 | Conclusion

This study found that a peak AEC of 1.5 × 10^9^/L offers a high sensitivity for clinically meaningful eosinophilia and need for eosinophilia-specific treatment. Higher absolute eosinophil thresholds offer greater specificity, but at the cost of reduced sensitivity and risk of missed cases. While these findings support considering an AEC threshold of 1.5 × 10^9^/L or higher as a criterion for hematology referral, especially in low-risk and asymptomatic patients, they should be interpreted cautiously and validated in larger, more diverse cohorts before broader adoption.

## Supplementary Material

supplementary material

Additional supporting information can be found online in the Supporting Information section. Table S1: Characteristics of patients with spontaneous normalization of eosinophilia prior to hematology evaluation. Table S2: Systemic treatment for secondary causes of eosinophilia. Figure S1: Time to normalization Kaplan–Meier curves.

## Figures and Tables

**FIGURE 1 |:**
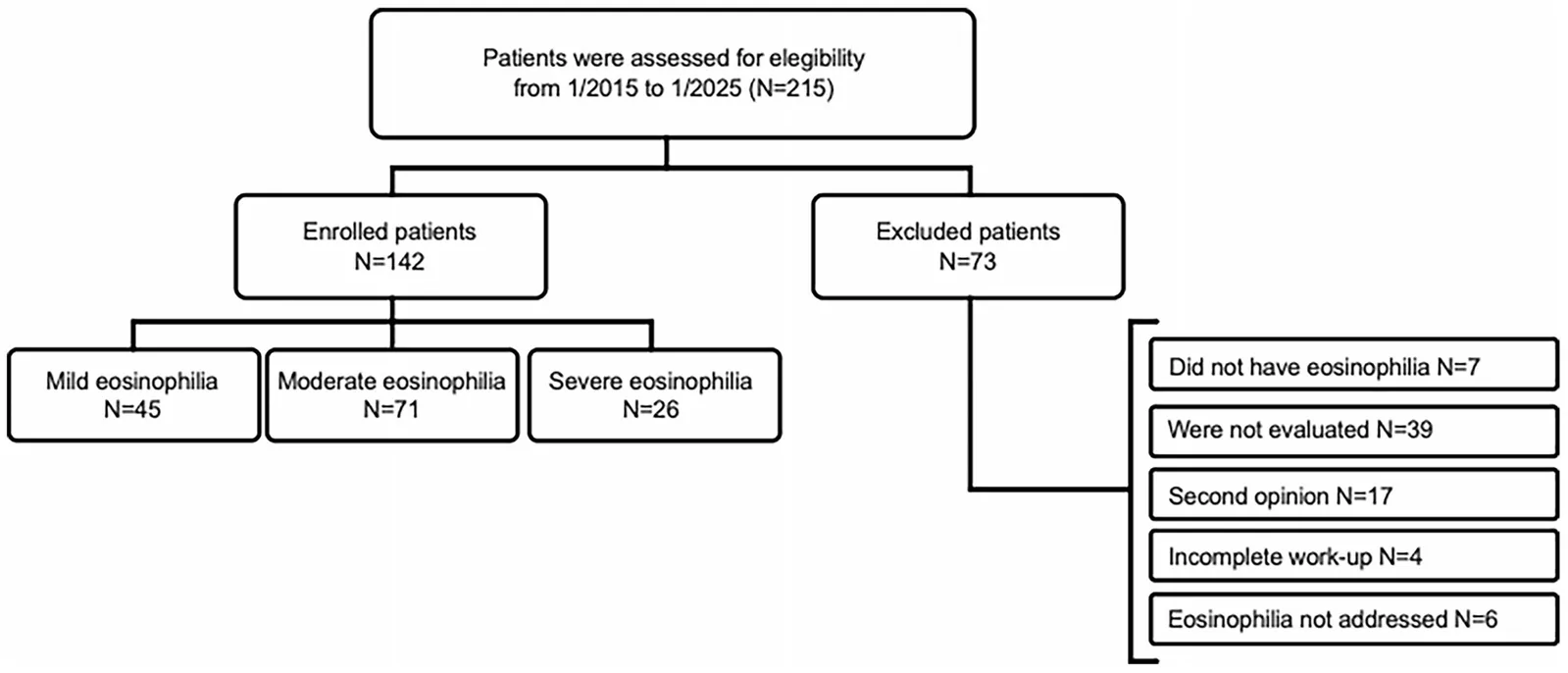
Inclusion and exclusion criteria.

**FIGURE 2 |:**
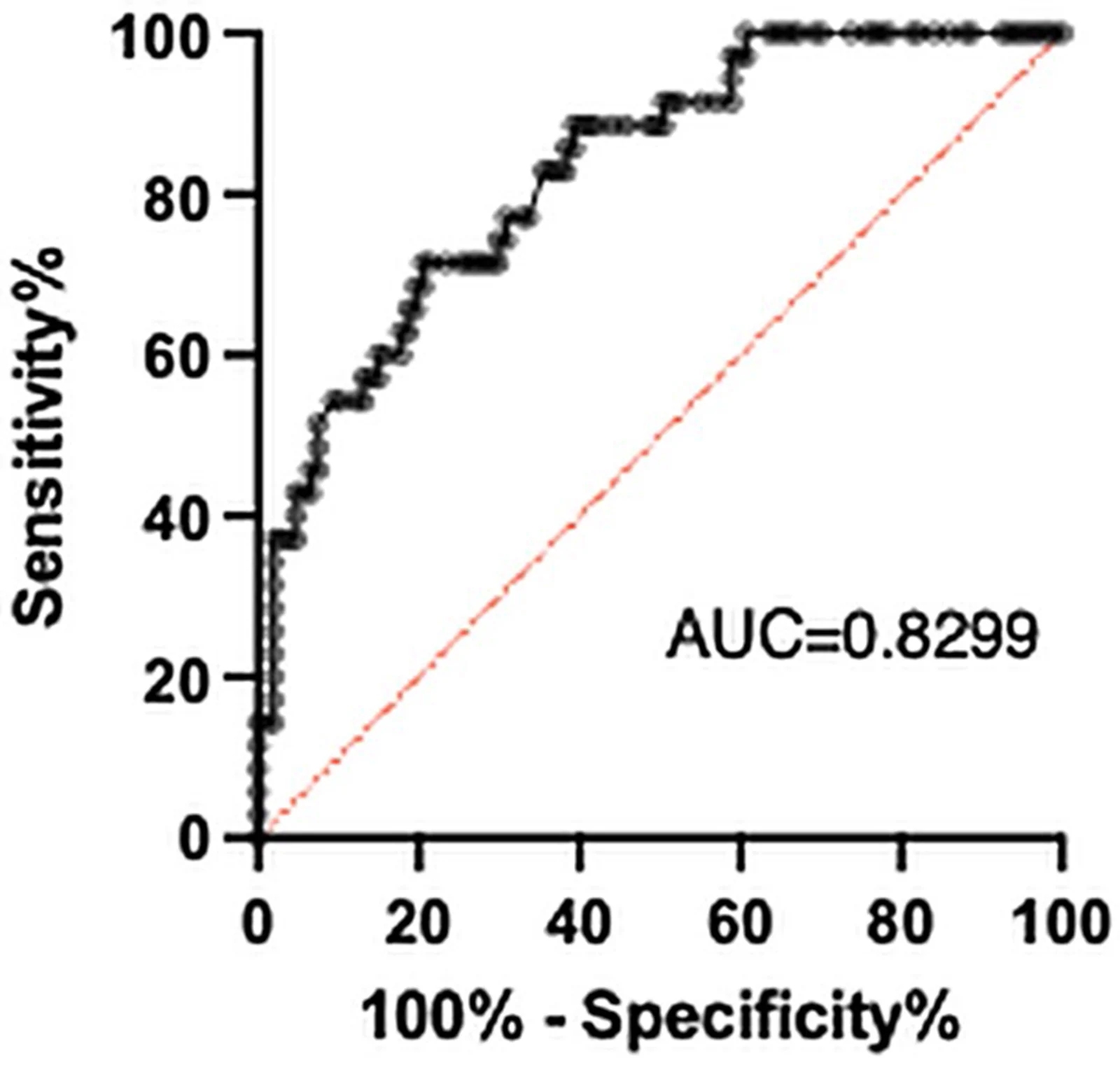
Receiver operating characteristic curve for clinically meaningful eosinophilia. AUC: area under the curve.

**FIGURE 3 |:**
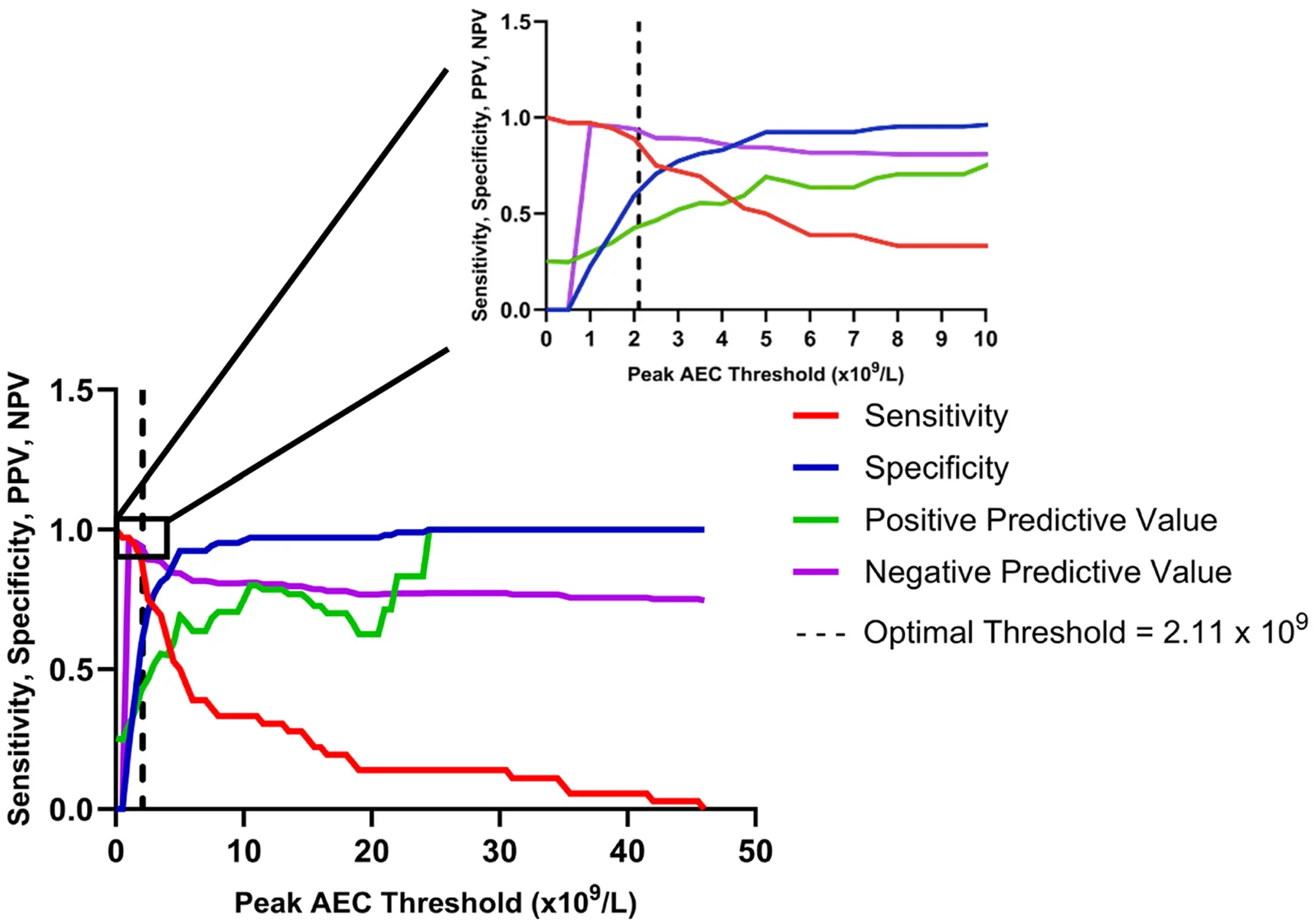
Sensitivity, specificity, and predictive values by absolute eosinophil count. AEC, absolute eosinophilia count; NPV, negative predictive value; PPV, positive predictive value.

**TABLE 1 |:** Patient characteristics.

	Number (*N* = 142)	%
Age (years)		
Mean ± SD	55.5 ± 17.1	
Sex		
Female	92	65
Male	50	35
Race		
Caucasian	116	81.7
African American	7	4.9
Asian/Asian Indian	12	8.5
Pacific Islander	1	0.7
Not reported	6	4.2
Ethnicity		
Not Hispanic	120	83.9
Hispanic	18	12.6
Not reported	4	2.8
Medical history		
Asthma	46	32.2
Autoimmune disease	17	11.9
Cancer	9	6.3
Normalization at time of appointment		
Yes	26	18.3
No	116	81.7
Peak absolute eosinophil count (×10^9^/L)		
500–1500	45	31.7
1500–5000	71	50
> 5000	26	18.3
Percent eosinophils (%)		
Mean ± SD, 27.7 ± 23.9		
Clinical diagnosis for eosinophilia		
Idiopathic	64	44.8
Hypereosinophilic syndrome	29	20.3
Asthma/pulmonary	14	9.8
Infection	11	7.7
Autoimmune	7	4.9
Drug reaction	7	4.9
Allergy	7	4.9
Gastrointestinal	3	2.1

Abbreviations: *N*, number; SD, standard deviation.

**TABLE 2 |:** Frequency of clinical diagnoses for hypereosinophilic syndrome (A), and clinically meaningful eosinophilia (B).

Clinical diagnosis	*N* = 29	%

*(A) Hypereosinophilic syndrome*		
HES-NOS	19	65.5
Autoimmune		
EGPA	3	10.4
IgG 4 disease	1	3.45
Malignancy/MPN		
T-cell lymphoma	1	3.45
ET	1	3.45
Overlap	2	6.9
Eosinophilic pneumonia	1	3.45
Chronic sinusitis	1	3.45

Clinical diagnosis	(*N* = 35)	%

*(B) Clinically meaningful eosinophilia*		
Hypereosinophilic syndrome	29	82.8
Eosinophilic pneumonia	3	8.6
EGPA	1	2.9
Drug related	1	2.9
Idiopathic	1	2.9

Abbreviation: *N*, number.

**TABLE 3 |:** Eosinophilia-specific treatment.

	Number (*N* = 34)	%
Prednisone	26	76.5
Mepolizumab	17	50.0
Hydroxyurea	2	5.8
Imatinib	2	5.8
Methotrexate	1	3.0
Pegylated Interferon	1	3.0

*Note:* Patients could have received more than one eosinophilia-specific treatment.

## Data Availability

The data underlying this study consist of identifiable patient-level information from a retrospective chart review and therefore cannot be shared publicly; de-identified data may be available from the corresponding author upon reasonable request and subject to institutional approval.
