# Supplementary material for: Redefining Hematology Referral Thresholds for Eosinophilia
Source: Eur J Haematol. Author manuscript; Available in PMC 2026 Oct 8. (PMC13649152; doi:10.1111/ejh.70152)
Supplement: Supplementary file 1 [file NIHMS2211972-supplement-supplementary_material.docx]

Supplemental Table 1: Characteristics of patients with spontaneous normalization of eosinophilia prior to hematology evaluation

|  | Number  (N=26) | % |
| --- | --- | --- |
| Age (years) |  |  |
| Mean ± SD | 52.5 ± 14.9 |  |
|  |  |  |
| Sex |  |  |
| Female | 16 | 61.5 |
| Male | 10 | 38.5 |
|  |  |  |
| Peak absolute eosinophil count (x10^9^/L) |  |  |
| 500-1500 | 21 | 80.8 |
| 1500-5000 | 5 | 19.2 |
| >5000 | 0 | 0 |
|  |  |  |
| Percent eosinophils (%) |  |  |
| Mean ± SD | 18.8 ± 17.0 |  |
|  |  |  |
| Clinical diagnosis for eosinophilia |  |  |
| Idiopathic | 15 | 57.7 |
| Infection | 4 | 15.4 |
| Allergy | 2 | 7.7 |
| Autoimmune | 2 | 7.7 |
| Drug reaction | 1 | 3.8 |
| Gastrointestinal | 1 | 3.8 |
| Asthma/pulmonary | 1 | 3.8 |

N: Number

SD: Standard deviation

Supplemental Table 2: Systemic treatment for secondary causes of eosinophilia

|  | Number  (N=26) | % |
| --- | --- | --- |
| Asthma/pulmonary |  |  |
| Dupilumab | 3 | 11.5 |
| Benralizumab | 2 | 7.69 |
| Prednisone | 1 | 3.85 |
| Allergy* |  |  |
| Omalizumab | 1 | 3.85 |
| Prednisone | 1 | 3.85 |
| Prednisone & methotrexate | 1 | 3.85 |
| Mycophenolate | 1 | 3.85 |
| Parasitic infection |  |  |
| Ivermectin | 9 | 34.6 |
| Paromycin | 1 | 3.85 |
|  |  |  |
| Gastrointestinal |  |  |
| Omeprazole | 1 | 3.85 |
| Autoimmune** |  |  |
| Adalimumab | 1 | 3.85 |
| Cyclosporine | 2 | 7.69 |
| Prednisone | 3 | 11.5 |
| Drug related |  |  |
| Prednisone | 1 | 3.85 |

Patients could have received more than one eosinophilia-specific therapy. Patients who received benralizumab and cyclophosphamide also received eosinophilia-specific treatment.

*Including mast cell activation syndrome and eosinophilic fasciitis.

**Including Gleich syndrome and eosinophilic granulomatosis with polyangiitis.

Supplemental Figure 1: Time to Normalization Kaplan-Meier Curves

**AEC: absolute eosinophilia count**
